# Revealing transcription factor and histone modification co-localization and dynamics across cell lines by integrating ChIP-seq and RNA-seq data

**DOI:** 10.1186/s12864-018-5278-5

**Published:** 2018-12-31

**Authors:** Lirong Zhang, Gaogao Xue, Junjie Liu, Qianzhong Li, Yong Wang

**Affiliations:** 1 School of Physical Science and Technology, Inner Mongolia University, Hohhot, Inner Mongolia 010021 China; 2 CEMS, NCMIS, MDIS, Academy of Mathematics and Systems Science, Chinese Academy of Sciences, Beijing, 100190 China; 3 School of Mathematical Sciences, University of Chinese Academy of Sciences, Beijing, 100049 China; 4 Center for Excellence in Animal Evolution and Genetics, Chinese Academy of Sciences, Kunming, 650223 China

**Keywords:** Transcription factor, Histone modification, Co-localization, Dynamics

## Abstract

**Background:**

Interactions among transcription factors (TFs) and histone modifications (HMs) play an important role in the precise regulation of gene expression. The context specificity of those interactions and further its dynamics in normal and disease remains largely unknown. Recent development in genomics technology enables transcription profiling by RNA-seq and protein’s binding profiling by ChIP-seq. Integrative analysis of the two types of data allows us to investigate TFs and HMs interactions both from the genome co-localization and downstream target gene expression.

**Results:**

We propose a integrative pipeline to explore the co-localization of 55 TFs and 11 HMs and its dynamics in human GM12878 and K562 by matched ChIP-seq and RNA-seq data from ENCODE. We classify TFs and HMs into three types based on their binding enrichment around transcription start site (TSS). Then a set of statistical indexes are proposed to characterize the TF-TF and TF-HM co-localizations. We found that Rad21, SMC3, and CTCF co-localized across five cell lines. High resolution Hi-C data in GM12878 shows that they associate most of the Hi-C peak loci with a specific CTCF-motif “anchor” and supports that CTCF, SMC3, and RAD2 co-localization serves important role in 3D chromatin structure. Meanwhile, 17 TF-TF pairs are highly dynamic between GM12878 and K562. We then build SVM models to correlate high and low expression level of target genes with TF binding and HM strength. We found that H3k9ac, H3k27ac, and three TFs (ELF1, TAF1, and POL2) are predictive with the accuracy about 85~92%.

**Conclusion:**

We propose a pipeline to analyze the co-localization of TF and HM and their dynamics across cell lines from ChIP-seq, and investigate their regulatory potency by RNA-seq. The integrative analysis of two level data reveals new insight for the cooperation of TFs and HMs and is helpful in understanding cell line specificity of TF/HM interactions.

**Electronic supplementary material:**

The online version of this article (10.1186/s12864-018-5278-5) contains supplementary material, which is available to authorized users.

## Background

Gene expression is known to be regulated by transcription factors (TFs) and histone modifications (HMs). To achieve precise regulation, those regulatory factors often work in a cooperative way. Physically, TFs and HMs tend to localize together at regulatory elements (promoter, enhancer, or insulator) in genome to achieve complex and accurate regulation of target genes [1–4]. For example, the initiation of transcription involves many protein-protein interactions among transcription factors, which bind to the promoter or enhancer and stabilize RNA polymerase [5–7]. In addition, recent studies have shown that histone modifications play significant regulation roles in the process of transcriptional initiation and elongation by interacting with transcription factors [8, 9]. Therefore, co-localization among TFs binding and HMs is critically important for understanding the precise control of gene expression [10, 11].

In general, there are two information sources useful to infer the cooperation among TFs and HMs. One is used to check the downstream effect on expression level of their target gene, which can be easily measured by microarray and RNA-seq. Previous studies have shown that TFs binding and HMs are predictive for gene expression in some model organisms [12, 13]. They found that histone modification levels and gene expression are very well correlated and only a small number of HMs are necessary to accurately predict gene expression in human CD4+ T-cells [14]. Using a Bayesian network, causal and combinatorial relationships among HMs and gene expression were investigated and some known relationships were confirmed [15]. Another information source is used to check the co-localization of TFs and HMs in chromatin, which can be measured by ChIP-seq technology [10]. Recently, Xie et al. [16] analyzed TF co-localization in human cells by a self-organizing map and revealed many interesting TF-TF associations and extensive change across cell lines. Furthermore, Zhang et al. [17] took long-range interactions into account and developed a new tool, named 3CPET, to infer the probable protein complexes in maintaining chromatin interactions. Taken together, a number of studies proved that TF/HMs’ cooperative interaction is important and can be investigated from various levels.

Here we argue that the localization data and downstream gene expression level should be integrated to predict high quality TF/HM interactions,because gene expression measured the results of TF/HM interactions while the upstream TF/HMs’ co-localization in genome provides the causal explanation for the effect. Integration of the two information sources, the direct co-localization in chromatin and the indirect effect on gene expression, is necessary and holds the promise to improve inference accuracy. With this solid base, the detailed interaction among TFs and HMs, its cell-line-specificity and diseases-specificity can be investigated.

Thanks to ENCODE Consortium, large scale data on whole-genome localization of protein–DNA binding sites [18, 19] and the absolute concentration of transcripts are available [20]. Particularly in some cell lines it provided the comprehensive ChIP-seq and matched RNA-seq data, for example genome-wide binding landscape of many TFs and HMs and target gene expression are available in human GM12878 and K562 cell lines (Additional file 1: Table S1). This allows us to investigate the relationship among TFs binding, HMs location, and gene expression in a systematic and quantitative manner. Meanwhile we can probe the dynamics of TF and HM co-localization in normal and cancer cell lines.

We propose a two-step integrative pipeline for ChIP-seq and RNA-seq. We first analyze and identify cooperation of TF and HM as well as the dynamics across normal and cancer cell lines. Then we investigate the regulatory potency of all these cooperations in gene expression process. To this end, we extracted signal peaks from the ChIP-seq data for 55 TFs and 11 HMs and the gene expression level from the RNA-Seq data in human GM12878 and K562 cell lines (Additional file 1: Table S2). The localization of 55 TFs and 11 HMs were analyzed in the upstream and downstream region of transcription start sites in the two cell lines. We observed three types of localization patterns, GM12878_rich_factor, K562_rich_factor, and unbiased_factor, based on their binding enrichment around TSS. Then, we compared the overlap ratio and the average overlap ratio of TFs’ binding or HMs in two cell lines. The results are further used to analyze potential cooperation of TFs and HMs. Finally, we build a SVM classifier to predict the highly and lowly expressed genes by utilizing the TF or HM association strength (TFAS) [21]. We found that two HMs (H3k9ac and H3k27ac) and three TFs (ELF1, TAF1, and POL2) are predictive with the accuracy about 85~92%. The highest prediction accuracy is 93% obtained by 66 factors model. Our research provides new insight for the cooperation of TFs and HMs on gene expression and is helpful for the study of the cooperation of various factors.

## Results

### The dynamics of TF and HM localization

**Fig. 1:**
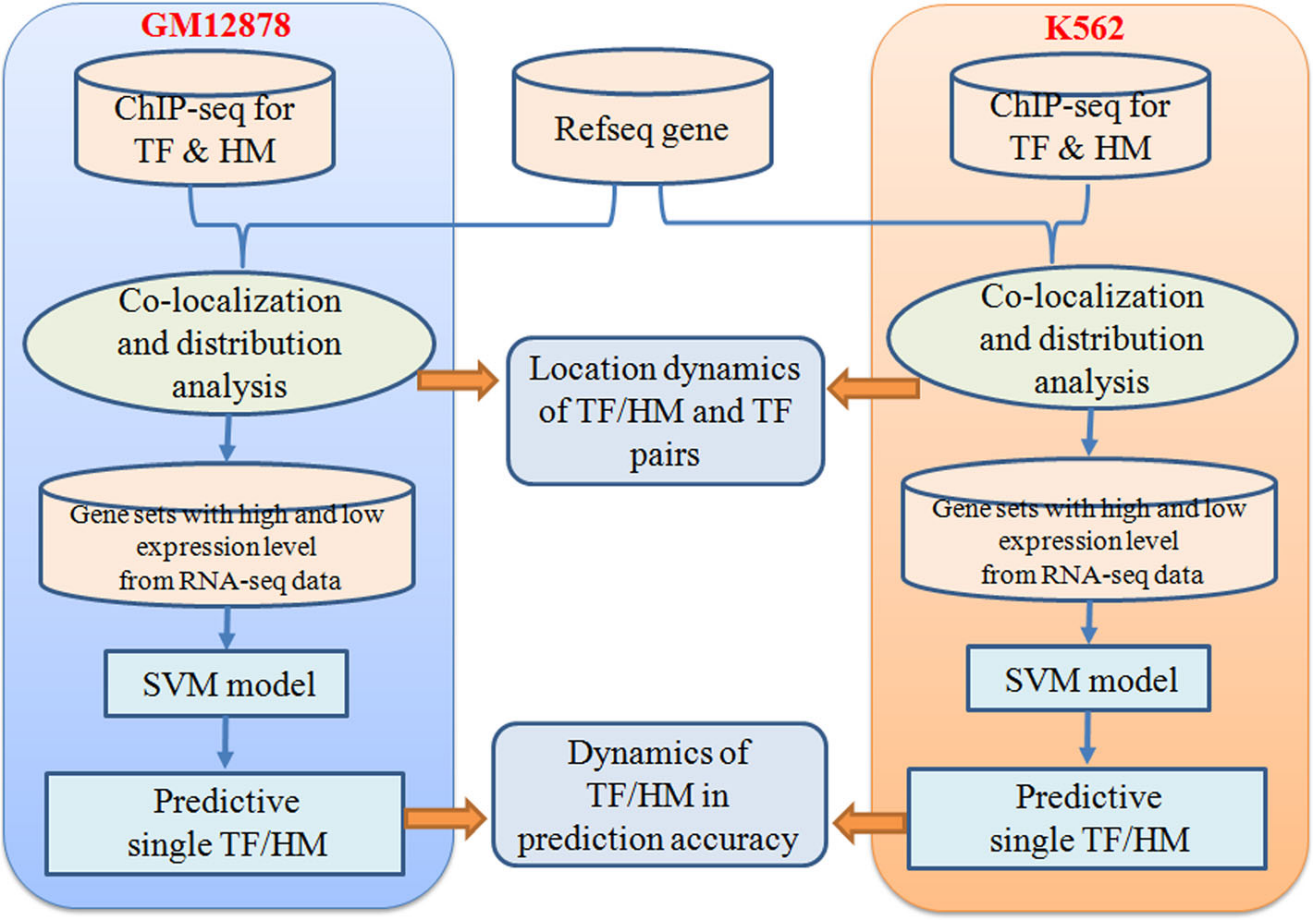
The two-step integrative pipeline to analyze matched ChIP-seq and RNA-seq data

We develop a two-step analysis pipeline (Fig. 1) to integrate ChIP-seq, RNA-seq, and genome annotation to pinpoint the unique roles of transcription factor and histone modification in biological processes and particularly their location at specific DNA region. Importantly we correlate TFs binding and HMs with gene expression level to detect reliable co-operations related with downstream effects. Crossing cell line comparison further indicate dynamic pattern of those co-operations.

**Fig. 2:**
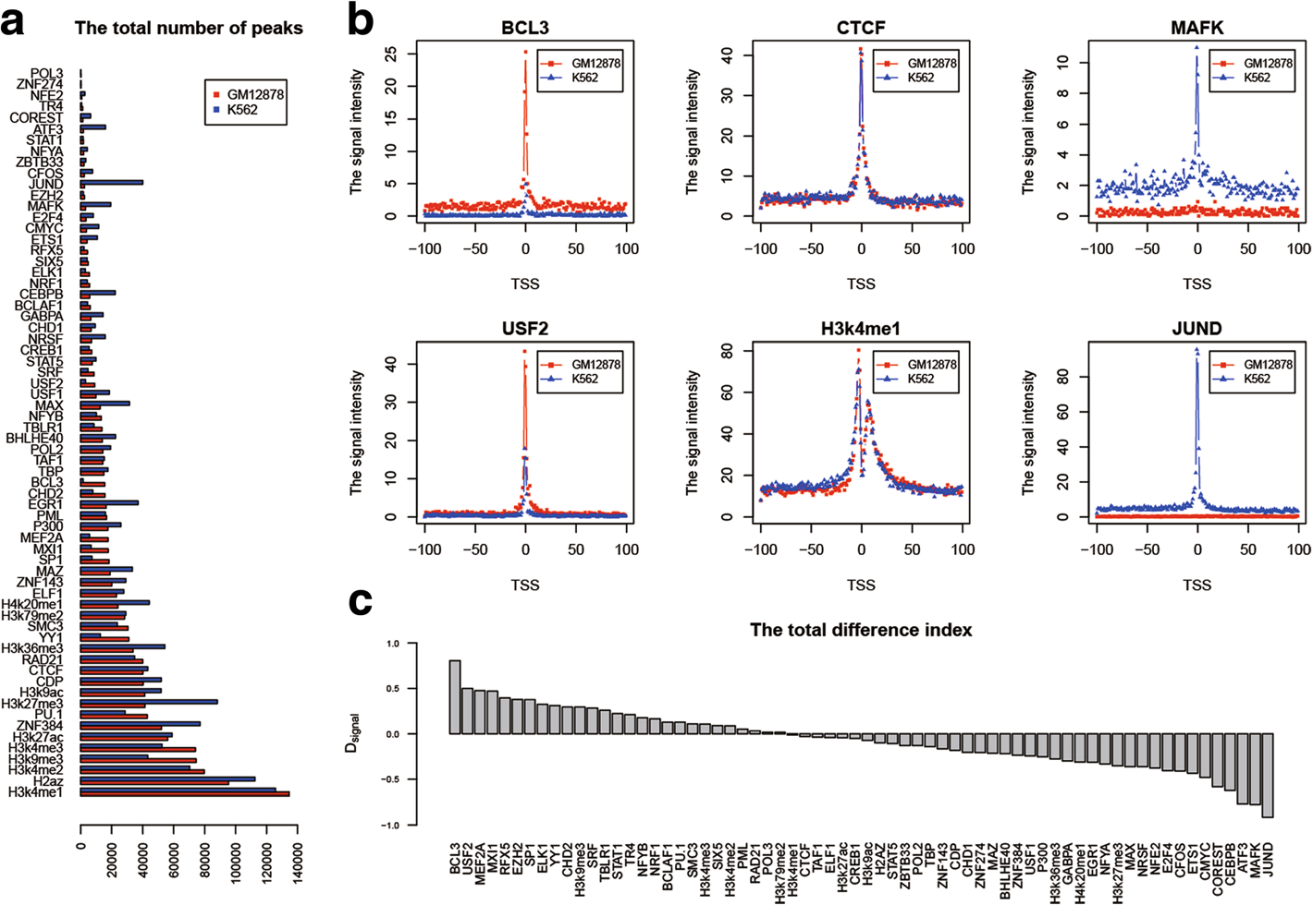
The dynamics of TF and HM localization between GM12878 and K562. **a** The peak numbers of 55 TFs and 11 HMs in two cell lines. The X-axis is the number of peaks, and the Y-axis represents the name of TF/HM. **b** The signal intensity of six factors in a 40 kb DNA region which was separated into 200 bins flanking TSS in two cell lines. Each bin is 200 bp in size. The X-axis is the relative position of bins, and the Y-axis is the signal intensity of a given TF/HM. **c** The total difference index of 55 TFs and 11 HMs between GM12878 and K562. The X-axis represents the name of TF/HM, and the Y-axis is the total difference index

Starting from the whole genome localization information produced by ChIP-seq experiment, we counted the peak number of 55 TFs and 11 HMs in two cell lines. As shown in Fig. 2a, the results indicated that the peak number is from 211/207 to 52,162/77,063 in GM12878/K562. H3k4me1 has a lot of peaks while POL3 has a few peaks. For some TFs or HMs, their peak numbers in two cell lines are quite different. If we set a and b as the total numbers of a given TF or HM in two cell lines, the values of |a ‐ b|/a + b for JUND, ATF3, BCL3, and MAFK are 0.88, 0.81, 0.81, and 0.72 respectively. And the maximum value of |a ‐ b|/a + b among 11 HMs is obtained by H3k27me3 with 0.36. On the other hand, the values of |a ‐ b|/a + b for POL3, PML, TAF1, and CTCF are 0.01, 0.02, 0.03, and 0.04 respectively. It shows that the numbers of peaks of these transcription factors are consistent in two cell lines.

We next check the signal features of TF binding and HM around TSSs, which are important for gene expression and regulation [13, 21, 22]. For 9555 genes, in two cell lines, we calculated the signal intensity of 55 TFs and 11 HMs in each of the 200 bins and obtained their distribution features in a 40 kb DNA region. It turned out that the signal peaks are concentrated in 4 kb region centered on TSS. The closer a bin gets to the TSS, the stronger the signal intensity of TFs or HMs. The distribution of six factors in two cell lines was shown in Fig. 2b. The signal intensities of CTCF and H3K4me1 show very similar distribution. But, some TFs or HMs have large variation such as BCL3, USF2, MAFK and JUND. Overall, there are three types of TF and HM based on their binding enrichment around TSS in two cell lines. We named them GM12878_rich_factor, K562_rich_factor, and unbiased_factor respectively for the follow-up study. Compared with HM, the variation of TF is larger. The results indicated that the signal intensity carries rich information to compare TFs binding and HM between normal and cancer cell lines.

To quantify the variation of TF binding or HM between two cell lines, we propose the total difference index *D*_*signal*_ and the ratio *f* to investigate the dynamics of TF or HM localization between the two cell lines (refer to eq. (2) and (3) in Methods section for the details). The rank of *D*_*signal*_ for all 66 factors shown in Fig. 2c can indicate the trend of all TFs’ and HMs’ variation between cell lines, and is used for analyzing their dynamic in two cell lines. The results showed some factors such as CTCF do not change much. Those factors mostly belong to the unbiased_factor set (32 factors) with 0.6 < *f* < 1.5 and −0.25 < *D*_*signal*_ < 0.2. This is consistent with the fact that CTCF works as a general transcription factor and is involved in many cellular processes, including transcriptional regulation, insulator activity, and regulation of chromatin architecture. BCL3 and JUND showed obvious difference. They belong to the GM12878_rich_factor set (15 factors) with *f* > 1.5 and *D*_*signal*_ > 0.2 and the K562_rich_factor set (19 factors) with f < 0.6 and *D*_*signal*_ <  − 0.25 respectively (Table 1). This demonstrates that our new index *D*_*signal*_ provides rich information to abstract TFs or HMs with cell line specificity for further investigation.

**Table 1:** Three sets of TF/HM based on their enrichment around TSS

GM12878_rich_factor			unbiased_factor			K562_rich_factor		
Factor	*f*	*D*_signal_	Factor	*f*	*D*_signal_	Factor	*f*	*D*_signal_
BCL3	9.28	0.81	NFYB	1.42	0.18	P300	0.60	−0.25
USF2	3.00	0.50	NRF1	1.39	0.16	H3k36me3	0.57	− 0.28
MEF2A	2.80	0.47	BCLAF1	1.29	0.13	GABPA	0.54	− 0.30
MXI1	2.77	0.47	PU.1	1.29	0.13	H4k20me1	0.53	− 0.31
RFX5	2.31	0.39	SMC3	1.24	0.11	EGR1	0.52	− 0.31
EZH2	2.21	0.38	H3k4me3	1.24	0.11	NFYA	0.50	− 0.33
SP1	2.21	0.38	SIX5	1.19	0.09	H3k27me3	0.48	− 0.35
ELK1	1.96	0.33	H3k4me2	1.19	0.09	MAX	0.47	− 0.36
YY1	1.90	0.31	PML	1.11	0.05	NRSF	0.47	− 0.36
CHD2	1.84	0.29	RAD21	1.07	0.03	NFE2	0.45	− 0.38
H3k9me3	1.83	0.29	POL3	1.03	0.02	E2F4	0.42	− 0.41
SRF	1.79	0.28	H3k79me2	1.03	0.02	CFOS	0.42	− 0.41
TBLR1	1.70	0.26	H3k4me1	0.97	−0.01	ETS1	0.39	− 0.43
STAT1	1.57	0.22	CTCF	0.94	− 0.03	CMYC	0.35	− 0.48
TR4	1.53	0.21	TAF1	0.93	− 0.04	COREST	0.26	− 0.58
			ELF1	0.92	− 0.04	CEBPB	0.23	− 0.62
			H3k27ac	0.91	− 0.05	ATF3	0.13	− 0.77
			CREB1	0.90	− 0.05	MAFK	0.13	− 0.77
			H3k9ac	0.86	− 0.07	JUND	0.04	− 0.92
			H2AZ	0.82	− 0.10			
			STAT5	0.81	− 0.11			
			ZBTB33	0.77	− 0.13			
			POL2	0.77	− 0.13			
			TBP	0.75	− 0.14			
			ZNF143	0.72	− 0.17			
			CDP	0.69	− 0.18			
			CHD1	0.66	− 0.21			
			ZNF274	0.66	− 0.21			
			MAZ	0.65	− 0.21			
			BHLHE40	0.64	− 0.22			
			ZNF384	0.62	− 0.24			
			USF1	0.61	− 0.24			

### The dynamics of TF-TF co-localization

**Fig. 3:**
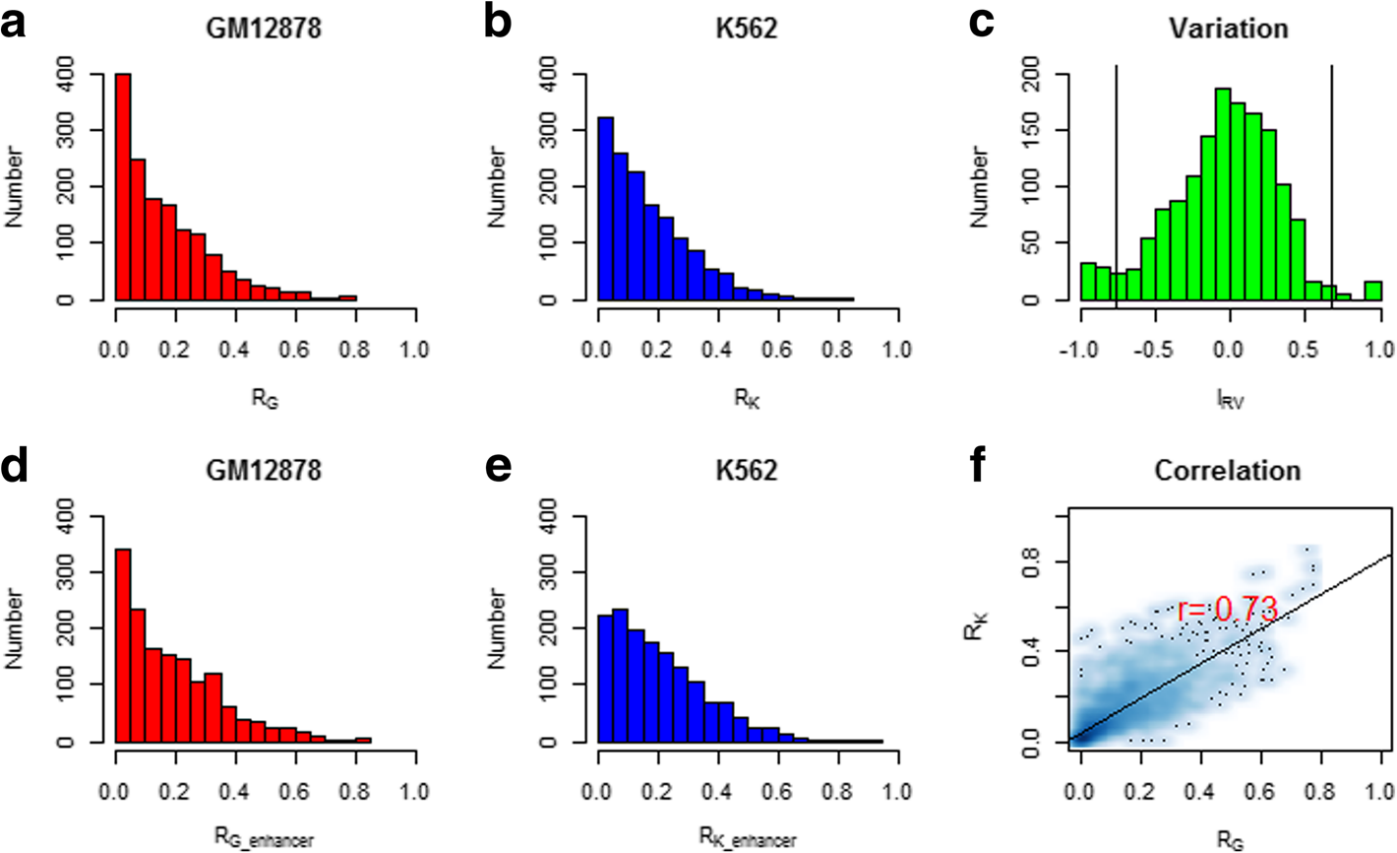
The overlap analysis of TF pairs in GM12878 and K562. The distribution of the overlap ratio for 1485 TF pairs in GM12878 ((**a**) for genome-wide and (**d**) for enhancer region) and K562 ((**b**) for genome-wide and (**e**) for enhancer region). The X-axis is the value of the overlap ratio, and the Y-axis is the number of TF pairs. **c** The distribution of the relative variation index *I*_*RV*_. The X-axis is the value of the relative variation index, and the Y-axis is the number of TF pairs. The left and right lines located the position with *μ* ± 2*σ*. And *μ* is the mean and *σ* is the standard deviation of the relative variation index *I*_*RV*_. **f** The scatter plot and the Pearson correlation coefficient of the overlap ratio for 1485 TF pairs between two cell lines. The X-axis and Y-axis are the overlap ratios of TF pairs in GM12878 and K562 respectively. Here R_*G*_ and R_*K*_ indicate the overlap ratios of TF pairs in GM12878 and K562 respectively

We next explore the cooperative interactions among TFs and HMs. In order to test the co-localization of TF and HM for genome-wide and enhancer regions, we calculated the overlap ratio R_*o*_ for all pairs of 55 TFs (Fig. 3a, b, d and e). Then, the Pearson correlation coefficient (PCC) of the R_*o*_ values for the 1485 TF pairs in two cell lines was calculated. The high correlation 0.73 (*p*-value< 2.2e-16) suggests that the co-localizations are overall conservative (Fig. 3d). The overlap ratio of RAD21 and SMC3 are 78.2% and 81.4% for genome-wide and enhancer regions separately in GM12878, and the value are 75.6% and 91.2% for genome-wide and enhancer regions separately in K562. For the combination of POL2 and TAF1, The overlap ratio are 76.1% and 80.7% in GM12878 and 84.6% and 94.6% in K562 separately. The results showed that there are stronger co-binding in enhancer regions for some TF pairs. In contrast, the overlap ratio between ZNF274 and any other TFs is almost zero which is may due to the less peaks of ZNF274 (233 in GM12878 and 305 in K562) according to the results of peak counting above. Based on the pairwise relationship, the combination patterns of three TFs with higher overlap ratio were obtained. POL2 + TAF1 + TBP (TATA Box Binding Protein) and Rad21 + SMC3 + CTCF show strong combination. The overlap ratios among them are more than 60%. By comparison, we found that their signal distribution around TSS was largely consistent (The total difference indexes are − 0.03, 0.03 and 0.11 for CTCF, RAD21, and SMC3). For the combination of Rad21 + SMC3 + CTCF, the results were consistent with previous works that CTCF is required to recruit cohesin complex members consist of Smc1/Smc3 heterodimers and two non-Smc subunits Scc1 (Rad21) and Scc3 to shared sites [16, 19, 23–25]. Furthermore, we obtained similar results for Rad21 + SMC3 + CTCF in Helas3, Sknsh, and Hepg2 cell lines. It demonstrates that higher overlap feature of the three TFs have certain conservation across cell lines (Table 2).

**Table 2:** The overlap ratios of TF combinations in five cell lines

TF combination	GM12878	K562	Helas3	Sknsh	Hepg2
RAD21:CTCF	0.777	0.779	0.686	0.856	0.757
RAD21:SMC3	0.781	0.756	0.805	0.846	0.657
CTCF:SMC3	0.745	0.672	0.701	0.854	0.637

Importantly, we found one strong Hi-C experimental data to support our finding in Table 2 and provide better understanding for the consistency of combination Rad21 + SMC3 + CTCF across five cell lines. Rao et al. used in situ Hi-C to probe the 3D architecture of genomes, constructing haploid and diploid maps of nine cell types [26]. The densest, in human lymphoblastoid cells, contains 4.9 billion contacts, achieving 1 kb resolution. They found that in GM12878 the vast majority of peak loci are bound by the insulator protein CTCF (86%) and the cohesin subunits RAD21 (86%) and SMC3 (87%). This result is consistent with our finding for CTCF+SMC3 + RAD21 combinations. This finding is also supported by numerous reports, using a variety of experimental modalities, that suggest a role for CTCF and cohesin in mediating DNA loops. Because many of these loops demarcate domains, this observation is also consistent with studies suggesting that CTCF delimits structural and regulatory domains [27–29]. They found that most peak loci encompass a unique DNA site containing a CTCF-binding motif, to which all three proteins (CTCF, SMC3, and RAD21) were bound [26]. They were thus able to associate most of the peak loci (6991 of 12,903, or 54%) with a specific CTCF-motif “anchor”. This supports that CTCF, SMC3, and RAD2 co-localization serves important role in 3D chromatin structure.

On the other hand, no matter how strong the total correlation is, the overlap ratios of some TF pairs show great changes. Let *R*_*G*_ and *R*_*K*_ be the overlap ratios of TF pairs in GM12878 and K562 respectively, the relative variation index *I*_*RV*_ between GM12878 and K562 is measure by (*R*_*G*_ − *R*_*K*_)/(*R*_*G*_ + *R*_*K*_ + *α*) (Fig. 3c). Here *α*=0.001 is added to avoid the case that *R*_*G*_ + *R*_*K*_ equals zero. The mean *μ* and the standard deviation *σ* of *I*_*RV*_ are − 0.05 and 0.36. And 90/1485 TF pairs are with significant variation falling outside *μ* ± 2*σ*.

By requiring the overlap ratio of TF pairs in both cell lines larger than the third quartile, we got 17 TF pairs (Table 3). For those TF pairs, their overlap ratios are with large changes between two cell lines. We found that there are 13 TF pairs related with JUND and only two TF pairs (BCL3:P300 and PML:USF1) have higher *R*_*G*_.

**Table 3:** TF pairs with cell line specificity

	TF combinations	*R*_*G*_	*R*_*K*_	*I*_*RV*_
1	ATF3:CEBPB	0.028	0.281	−0.821
2	ATF3:JUND	0.002	0.449	−0.991
3	ATF3:P300	0.032	0.358	−0.838
4	BCL3:P300	0.366	0.071	0.676
5	CMYC:JUND	0.007	0.251	−0.945
6	EGR1:JUND	0.024	0.300	−0.849
7	ELF1:JUND	0.015	0.341	−0.918
8	ETS1:JUND	0.013	0.263	−0.907
9	GABPA:JUND	0.002	0.311	−0.986
10	JUND:MAX	0.027	0.465	−0.891
11	JUND:MAZ	0.041	0.488	−0.844
12	JUND:NRSF	0.013	0.254	−0.903
13	JUND:POL2	0.016	0.347	−0.914
14	JUND:TAF1	0.031	0.315	−0.823
15	JUND:ZNF143	0.005	0.291	−0.968
16	JUND:ZNF384	0.039	0.346	−0.798
17	PML:USF1	0.280	0.000	1.000

On the other hand, by calculating the TFAS value of 55 TFs based on their signal peaks in 40 kb region centered on TSS, we obtained the PCC values of TF pairs to explore its interaction tendency. The POL2 + TAF1 + TBP and Rad21 + SMC3 + CTCF combinations display higher PCC values. The results are consistent with the above analysis (Table 4).

**Table 4:** TF pairs with top 10 PCC in GM12878 and K562

Cell line	Index	RAD21/CTCF	RAD21/SMC3	CTCF/SMC3	TAF1/PML	TAF1/POL2	POL2/PML	TBP/POL2	TBP/TAF1	MAX/MXIL	MAX/CMYC
GM12878	*R*_*o*_	0.777	0.781	0.745	0.708	0.761	0.776	0.612	0.577	0.731	0.514
	PCC	0.787	0.865	0.760	0.735	0.766	0.776	0.710	0.708	0.731	0.514
K562	*R*_*o*_	0.779	0.756	0.672	0.645	0.846	0.692	0.747	0.736	0.555	0.705
	PCC	0.781	0.830	0.738	0.760	0.828	0.759	0.799	0.819	0.554	0.705

**Fig. 4:**
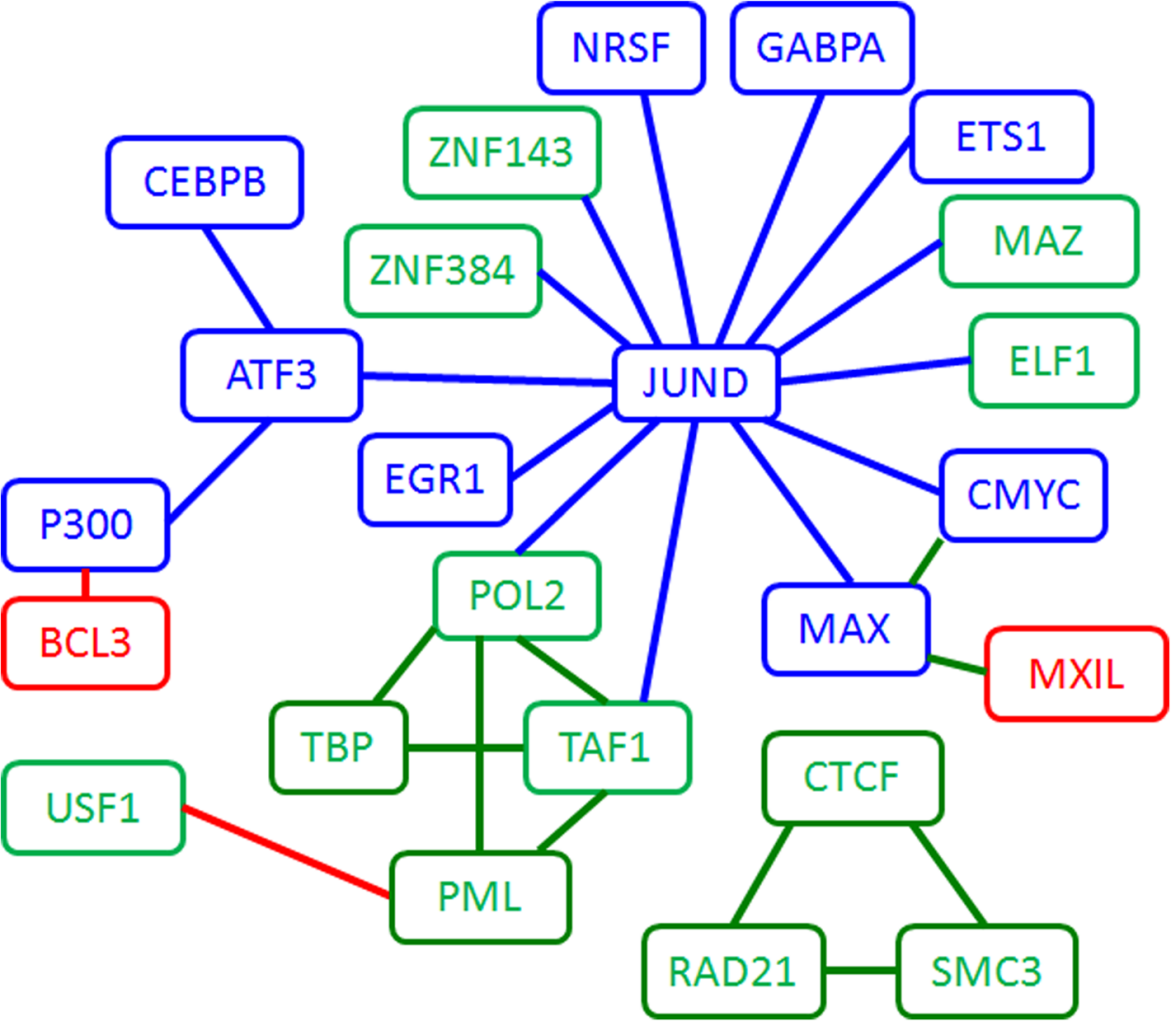
The interaction network among TFs. The node color labels the TF type (Red: GM12878_rich_factor; Blue: K562_rich_factor; Green: unbiased_factor) and the edge color indicate the specificity of TF pairs in different cell lines (Red: GM12878_specificity_TF pairs; blue: K562_specificity_TF pairs; Green: unbiased_TF pairs)

By choosing a threshold, we obtained a TF interaction network as shown in Fig. 4. We use different node colors to label the GM12878_rich_factor, K562_rich_factor, and unbiased_factor. The edge colors indicate the specificity in different cell lines (GM12878_specificity_TF pairs, K562_specificity_TF pairs, and unbiased_TF pairs). The network shows that JUND serves as a hub in K562 and plays important roles in cancer by interacting with other TFs. It’s also interesting that JUND cooperates with ATF3 and together working with chromatin factors P300 and CEBPB. While in GM12878, BCL3 alone works with P300 and may guide the chromatin factor to activate regulatory regions. Comparing with the giant complex in K562, GM12878 uses a very different strategy. CTCF + RAD21 + SMC3 and POL2 + TBP + TAF1+ PML are tight clusters in the network and required in both cell types. This TF and chromatin factor co-operation is consistent with previous studies that HMs regulate gene transcription by modulating local chromatin state and thereby changing the binding status of TFs within gene regulation regions [13, 30]. And the analyses based on the experimental data indicated that distinct HM patterns appear around TF binding sites, and the ChIP-seq signals of TFs binding and HMs are highly predictive of each other [30–32]. Based on the clique like interaction, we can predict that TBP and PML cooperate.

Next we add the HMs in the cooperation analysis. Based on the peak signal data of 11 HMs, the overlap ratios between 11 HMs and 55 TFs were calculated for GM12878 cell line. The results showed that there was consistency for the overlap features of 11 HMs with TFs. But the overlap ratio of the same HM with different TFs had large variations (Additional file 1: Figure S1). Part of HMs (H3K9ac and H3K79me2) obtained higher overlap ratio greater than 50%, which indicated close relationship between these HMs and TFs. The studies in K562 give us consistent conclusions.

### The average overlap ratio of TFs and HMs

**Fig. 5:**
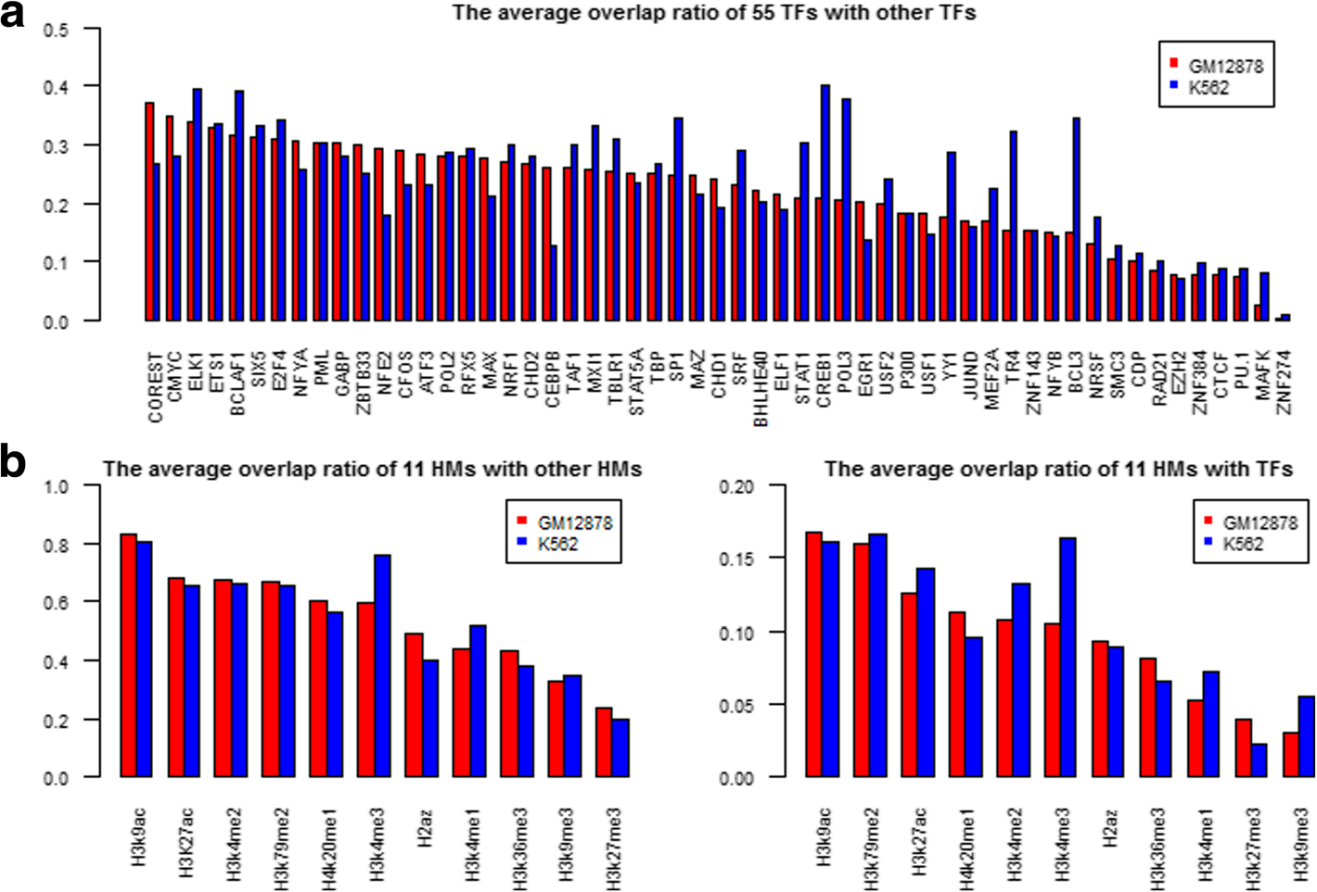
The average overlap ratio of TFs and HMs. **a** The average overlap ratio of 55 TFs in two cell lines. **b** The average overlap ratio of 11 HMs with other HMs (left) or 55 TFs (right). The X-axis represents the name of TFs/HMs, and The Y-axis represents the average overlap ratio

To get a clear understanding of the potential cooperativity between a certain TF and other TFs, we defined a new parameter *R*_*av*_ named the average overlap ratio. For each TF or HM, we calculated its *R*_*av*_ and found that the *R*_*av*_ values of 66 factors presented clear divergence in a cell line. It is a range from 40 to 3%. Among them, COREST, CMYC, ELK1, ETS1, and BCLAF1 are the top 5 TFs with the higher *R*_*av*_ in GM12878, and CREB1, ELK1, BCLAF1, POL3, and BCL3 are the top 5 TFs in K562 (Fig. 5a), with two common factors ELK1 and BCLAF1. Next, we found that the average overlap ratios of some TFs have significant variation between GM12878 and K562. The *R*_*av*_ values of each TF in the two cell lines are roughly consistent for most TFs, with the exception of a few TFs including BCL3 and CREB1. For example, in K562, CREB1 is the TF with the top location in the *R*_*av*_ list, but in GM12878 its relative location is ranked in 33. Both of them are related with Leukaemia [33]. BCL3 gene is a proto-oncogene candidate which is identified by its translocation into the immunoglobulin alpha-locus in some cases of B-cell leukemia. And CREB (cyclic AMP response element-binding protein) is a transcription factor associated with neoplastic myelopoiesis by regulating RFC3 (Replication factor C3) expression [34]. The results indicated that the TF combination patterns have specificity in GM12878 and K562 cell lines.

On the other hand, for the TF pairs with higher overlap, its average overlap ratio is lower. For example, no matter how big the total peak number or the overlap ratio for CTCF is, its average overlap ratio is always the lowest. The average overlap ratio of CTCF is about 8%, although the combinations of CTCF with Rad21 or SMC3 have a higher overlap ratio about 70% in both cell lines. In opposite, some TFs have lower overlap ratio, but they have higher average overlap. For instance, the overlap ratios of ATF3 are less than 2% with CDP, EZH2, JUND, POL3, PU.1, RAD21, and ZNF274, however its average overlap ratios are 28.4% in GM12878 and 23.02% in K562. The average overlap ratio of TFs provides a new clue about its overall interaction capability with other TFs.

TF and HM are two types of critical factors that coordinately regulate gene transcription. As a consequence, TF-binding and histone-modification are often highly correlated in TSS proximal regions. Based on the same definition, we calculated the average overlap ratio of a HM with other 10 HMs as well as 55 TFs in two cell lines (Fig. 5b). The results indicated that HMs related with gene silencing such as H3K27me3 and H3K9me3 have lower *R*_*av*_, but ones related with gene activating have higher *R*_*av*_ such as H3K9ac and H3K27ac. This results show a certain coincide with Bieberstein’s studies [35]. Their researches presented that the activating histone modifications H3K4me3 and H3K9ac mapped to first exon-intron boundaries to help recruit general transcription factors (GTFs) to promoters [36]. It is possible that the marks changes chromatin states by affecting the affinity between histone and DNA, and further produce an effect on the TF binding with DNA. Among them, H3K9ac exhibit a maximum *R*_*av*_ which is 80%. That provides a great chance for histone modification to model TF binding affinities. As a result, HMs could help the prediction of TF binding sites [31].

### Pinpoint TFs and TF-TF interaction with gene expression

We next pinpoint TFs and TF-TF interaction to predict their downstream effect, i.e., predicting gene expression level with TFs or HMs. As we know, gene expression has cell line or tissue variation. The prediction of gene expression level in a particular tissue and its dynamics across tissues are very important for the study of expression regulation. Here we look at the relative contribution of each factor in more details in order to understand gene regulatory mechanism. We constructed a classification model based on SVM to examine the relative importance of each individual factor [37]. Based on the FPKM (fragments per kilobase of exon per million fragments mapped) values [20], all of 9555 genes were classified into two categories with high or low expression level. Then, the relative importance can be represented by the predicting capability for discriminating gene categories as high or low expression level in human genome. In each cell line, the SVM model was built for each TF or HM with its association strength (TFAS) as inputs and gene’s group (high or low expression level) as outputs.

Firstly, we constructed a SVM model for the identification of gene expression level using each TF or HM as the single predictor. The prediction accuracies were shown in Table 5. Strikingly, most TFs alone can predict gene expression levels with fairly high accuracies. By direct comparison, TFs and HMs presented different capability for predicting gene expression level. We found that some factors such as H3k9ac, H3k27ac, ELF1, TAF1, and POL2 were significantly more predictive than other factors. These factors mostly possess transcriptional activation function and have more peaks. These TF bindings are essential for transcriptional initiation of most promoters, and therefore it makes sense that their binding signals have the highest predictive capabilities. In contrast, other factors such as MAFK, POL3, ZNF274, EZH2, NFE2, and TR4 were significantly less predictive. Those factors generally have lesser peaks and tend to have specific or complex functions. It is expected that these TFs such as POL3 are less predictive because they are involved in initiating transcription of only a small fraction of promoters. This provides a clue that the factors with more peaks are related with cell type non-specific genes and the factors with less peaks are related with cell type specific genes. Furthermore, the TFs or HMs with low average overlap ratio may be associated with expression of cell type specific genes. In general, Enrichments (with more binding peaks) of HM or TF at transcription start site are positively related to its high predictability.

**Table 5:** The prediction accuracies of gene expression level for 66 factors in two cell lines (Acc values)

TF/HM	GM12878(%)	K562(%)	TF/HM	GM12878(%)	K562(%)	TF/HM	GM12878(%)	K562(%)
H3k9ac	89.18	90.75	PU.1	71.52	70.87	STAT5	60.15	60.21
ELF1	88.05	86.77	TBLR1	70.45	63.27	RAD21	59.98	58.56
TAF1	87.67	90.18	GABPA	70.01	77.88	H3k27me3	59.61	62.45
POL2	86.65	91.77	NRF1	69.46	65.09	NRSF	59.00	63.88
H3k27ac	85.96	91.69	NFYB	69.07	63.04	H4k20me1	58.16	62.35
MXIL	85.31	68.23	SRF	68.08	61.18	CREB1	57.97	64.88
YY1	84.03	82.17	CHD1	68.02	76.89	SMC3	57.91	55.25
MAZ	83.99	87.94	ELK1	66.72	59.98	CFOS	57.43	61.66
PML	83.21	81.25	USF1	66.62	69.46	CEBPB	56.82	68.67
EGR1	82.21	84.14	USF2	66.43	56.13	H3k9me3	56.32	60.93
H2az	82.17	81.88	ZNF143	65.97	68.48	ZBTB33	56.09	57.30
CHD2	79.97	70.11	H3k36me3	65.22	72.23	NFYA	55.90	60.00
TBP	79.89	88.89	MEF2A	64.92	56.43	STAT1	54.96	52.43
SP1	79.80	68.31	P300	64.80	72.58	ATF3	54.58	72.60
ZNF384	79.70	87.00	SIX5	64.78	62.24	Corest	53.35	57.81
MAX	79.57	88.32	CMYC	64.02	76.87	EZH2	52.80	51.26
H3k4me2	77.86	86.29	ETS1	62.93	78.42	NFE2	52.20	52.64
H3k4me3	77.15	88.09	BCLAF1	62.91	61.74	TR4	52.09	52.16
CDP	76.52	83.09	E2F4	62.20	78.21	JUND	51.84	79.78
H3k79me2	75.45	78.82	BCL3	61.55	52.49	MAFK	50.86	59.21
H3k4me1	73.04	82.71	CTCF	61.47	60.36	POL3	50.19	50.15
BHLHE40	71.95	77.21	RFX5	61.09	54.12	ZNF274	50.04	50.06

Next, the total 66 association strengths of 55 TFs and 11 HMs were used to predict gene expression level and the highest classification accuracy is achieved as 92.2% and 93.7% for GM12878 and K562 respectively (Table 6). We found that the 66 factors model could identified genes with a slightly higher accuracy than the single factor models. The accuracies are 3% and 1.9% more than the highest prediction accuracies with single factor. The high prediction accuracies across two cell lines suggested the strong correlations between gene expression level and TF binding or HMs in two considered cell conditions. But, the limited improvement also illustrated that there are a certain extent redundancy between factors which means they share a similar amount of information for “predicting” gene expression level.

**Table 6:** The prediction accuracies of gene expression level in two cell lines

Input	GM12878			K562		
	Sn	Sp	Acc	Sn	Sp	Acc
The total 66 factors	95.0%	89.4%	92.2%	94.5%	93.0%	93.7%
The top 10 factors with *D*_*Acc*_ > 0	90.4%	88.7%	89.5%	83.9%	91.8%	87.8%
The bottom 10 factors with *D*_*Acc*_ > 0	68.4%	91.6%	80.0%	86.6%	91.5%	89.1%

Based on the prediction results of single factor, for any TF or HM, we defined a prediction difference index between two cell lines.

$$ {D}_{Acc}=\frac{Acc^G-{Acc}^K}{Acc^G+{Acc}^k} $$

**Fig. 6:**
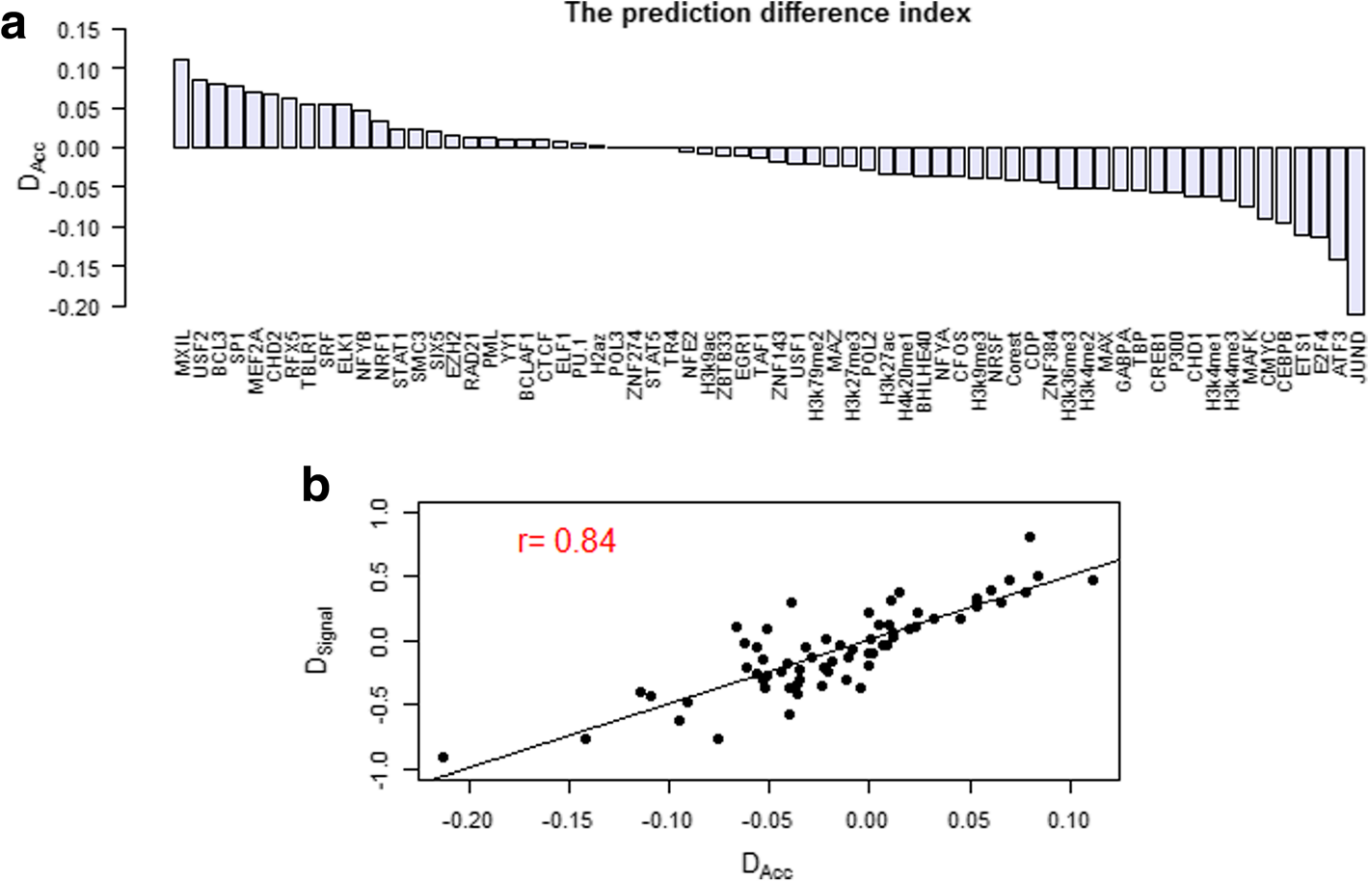
The prediction difference index for TFs/HMs. **a** The rank list of the prediction difference index *D*_*Acc*_ for 66 factors. The X-axis represents the name of TF/HM, and the Y-axis represents the prediction difference index. **b** The correlation properties between the prediction difference index *D*_*Acc*_ and the total difference index *D*_*signal*_. The X-axis is the prediction difference index, and the Y-axis is the total difference index

Where *Acc*^*G*^and *Acc*^*K*^ are the prediction accuracies of a given TF or HM in GM12878 and K562 separately. The rank list of *D*_*Acc*_ are shown in Fig. 6a.

We then extracted the factors with the top ten *D*_*Acc*_ (*D*_*Acc*_ > 0) or the bottom ten (*D*_*Acc*_ < 0) as inputto constructed the SVM model of expression level prediction. The prediction accuracies of the top ten are 89.5% and 87.8%, and the bottom ten are 80.0% and 89.1% respectively in two cell lines. As shown in Table 5, the prediction performances of the top ten TF and HM signals almost achieved the highest accuracies which are ~ 2.7% and ~ 4.6% lower than the performance by the full factors model. And this result is even lower than the prediction of some single factor.

In Fig. 6, we found that the prediction difference index *D*_*Acc*_ is consistent with the total difference index *D*_*signal*_ which is a parameter represented the dynamic variation of a TF binding or HM between two cell lines. To further demonstrate the relationship between *D*_*signal*_ and *D*_*Acc*_, we then calculated the Pearson correlation coefficient as 0.84 (Fig. 6b). The results directly indicated that the dynamic variation of TF binding or HM distribution around TSS between two cell lines is positively related to its prediction power difference of gene expression level. Meanwhile, the results also illustrated that the predicting power of a TF/HM would present obvious difference if its binding has dynamic variation around TSS between two cell lines. We suppose these factors with great dynamic variation should be strongly associated with cell line specific regulation. For example, JUND may be related with specific vital process in K562. On the other hand, the factors with higher predictive capability such as H3K9ac and H3k27ac barely appeared the variation among cell lines. In general, they should take part in the basic regulation processes.

## Discussions

### Interaction of TFs and HMs

Co-occupancy of TF binding is a key mechanism for fine regulation of gene expression. However, there are no reliable approach for computationally measuring the degree of TF-TF cooperation and quantitatively modeling the dynamic variation between cell lines. We here introduced a set of statistical indexes to investigate the degree of TF-TF or TF-HM genome-wide overlap in TSS region. The overlap ratio of TFs provides a quantitative parameter for measuring the degree of TFs interaction. The higher the value is, the greater the chance of their interaction to regulate gene expression. On the contrary, TFs with low overlap ratio should be mutually-exclusive. We obtained some TF combinations confirmed by previous experiments, also found new combinations for further experiments. In addition, dynamics among cell lines provided an approach to study the dynamic of TFs or HMs cooperation in the regulation process of gene expression. We suppose that their interactions of TF combinations with little variation are conserved in two cell lines. In fact, the prediction of TF binding site by histone marks, or vice versa, substantially depends on their higher co-occupancy. Also it gives us a clue for information redundancy analysis of TFs or HMs in predicting of gene expression level. We can extract a set of TFs or HMs based on the overlap analysis for predicting models.

Meanwhile, the analysis of dynamic or conservation for the combination of TF pairs is able to capture the vast complexity of colocalization patterns, resulting in identification of many previously known interactions. For example, we identified ATF3:JUND as a K562-specific combination. In fact, the ATF3/JUND heterodimer preferentially binds to an AP-1-like site and are most likely the important mediators of the response because overexpression of JUND [38]. On the other hand, we found the conservative combination CTCF:Rad21 which act as host cell restriction factors for Kaposi’s sarcoma-sssociated herpesvirus (KSHV) lytic replication by modulating viral gene transcription [39]. In addition to some confirming known combinations, we also found additional colocalization patterns that have not been previously documented. These may exist as entirely novel combinations for further confirmation. Our results provide many insights into TF colocalizations that define the regulatory code of humans.

### The relative importance of TFs and HMs for classification

The accurate regulation of gene expression is a complex process and many TFs and HMs participated. In previous studies, it has been shown that TF binding and histone modification are predictive for expression levels of mRNA transcripts in some cell lines. However, these studies have been limited to a limited number of TF or HM data at that time. In 2010, Karlic et al. [14] systematically analyzed 38 HM and they only used the numbers of tags for each histone modification or variant in 4 kb surrounding the TSSs. They did not consider the distance between HM and TSS. In our paper, not only HM but also TF association strength (TFAS) that integrated all the peak intensity of a TF/HM by considering their proximity to a gene is used to predict gene expression level. Next, we built the SVM model with single TF or HM to predict binary classification as high or low gene expression and evaluated the performance using accuracy. But Ouyang et al. [21] and Cheng et.al [13] employ the correlation to evaluate the predictive power by calculating the Pearson correlation coefficient (PCC) value between the observed gene expression values and the predicted values. Our method is more straight-forward to capture the main signals with comprehensive data.

In particular, the relative importance of these factors in the regulation of gene expression is still under debated. Furthermore, it is a long way to go to precisely quantify the expression level of each gene. In this study, we avoid this challenge by an alternative way to classify the high and low expression genes. We constructed a SVM model with single TF or HM and focus on investigating the relative contribution of TF binding or HMs in the prediction of gene expression level. By listing TFs and HMs based on the predicting power, we can understand their potential capability in gene regulation. The results show that the prediction accuracies vary significantly with the substitute among HMs and TFs. Furthermore, our results suggest that two types of HMs (H3k9ac and H3k27ac) with activation expression function and three TFs (ELF1, TAF1, and POL2) are predictive for gene expression with the accuracy about 85~92%. And the active TFs have higher prediction power than the repression TFs. And the highest predictive accuracy was achieved for gene classification by the 66 factors model.

We compare the predictive difference of a certain TF or HM between two cell lines. The results indicated that some factors change dramatically. We have previously shown that the single factor model for gene expression prediction is cell line specific. The best prediction accuracies are achieved by H3K9ac in GM12878 but POL2 in K562. In addition, TFs and HMs show different relative importance in different cell lines. A TF might be active and exhibit significant influence on gene expression in K562, but inactive with little effect on gene expression in GM12878. For example, JUND shows a relatively stronger effect on gene expression in K562 than in GM12878 while MXIL shows the opposite trend. Based on the correlation analysis of *D*_*signal*_ and *D*_*Acc*_, we found that the variation of predicting power is closely related with its distribution dynamic variation around TSS in two cell lines. And those TFs with simplex function always present higher predictive capability, for instance, active factors such as H3K9ac, ELF1, TAF1, POL2, H3K27ac, EGR1, or repressive factors such as MXI1 and CDP. But the prediction power of the TFs with complex or bidirectional functions such as ATF3, CTCF, and SRF is weak.

### Simplified model with six factors

For a few TFs and HMs with higher predictive power, we found their total difference indexes *D*_*signal*_ are the lowest, and their overlap ratio and average overlap ratio are high. For example, the prediction accuracies of POL2, TAF1, and TBP are 86.6%, 87.6%, and 79.9% in GM12878 and 91.8%, 90.2%, and 88.9% in K562. Meanwhile,the TFs with highest overlap ratio but lower average overlap ratio have moderate prediction power such as Rad21, SMC3, and CTCF. Their prediction accuracies are 60.0%, 57.9%, and 61.5% in GM12878 and 58.6%, 55.3%, and 60.4% in K562.

Then, a six factors model including POL2, TAF1, PML, ELF1, H3K27ac, and H3K9ac was constructed. The six factors chosen have transcriptional activation function and higher predictive power. The prediction accuracies are 92.0% and 93.3% and pretty close to the prediction accuracy of all 66 factors. Adding other TF/HM features cannot improve the prediction power of gene expression level. The results give us an idea that some major factors are the most useful in predicting of gene expression level. This observation is consistent with the results in [21] that only a handful of TFs’ binding can explain the large percentage of expression variance. From our study, we can extract key TFs or HMs based on the analysis of the overlap and average overlap ratio to predict gene expression level.

### Future extension with cis-regulatory element annotation

We acknowledge the limitation that we mainly focus on the cooperation in trans level. It’s well known that the cis-regulatory elements (specifically enhancer) are important to work together with trans-element (TF and HM) to precisely determine the downstream gene expression. Here we focus on the complexity at trans level, i.e., the combinatorial effect of TF and HM by checking their co-localization in regulatory element and downstream gene expression effect. We implicitly consider the enhancer by looking at the distal binding peaks of TF and HM and summarize the binding strength. However, we didn’t look at the specific “enhancer” region together with co-localized TFs/MHs, which will provide more detailed and enriched information. Furthermore, we simplified the multiple to multiple mappings between regulatory regions to target genes. We will extend the current work to TF, HM and regulatory element cooperations. In future we will also integrate some new data types, for example ATAC-seq and Hi-C/HiChIP, and hold the promise to provide binding profiles for many TFs once and high resolution regulatory element-gene association.

## Conclusions

In summary, we analyzed the distribution and overlapping state of TF and HM and obtained three types of TF and HM (GM12878_rich_factor, K562_rich_factor and unbiased_factor) based on their enrichment around TSS in two cell lines. We calculated the overlap ratio of 1485 TF pairs to test the genome-wide co-localization in two cell lines. The correlation analysis indicated that their co-localizations are overall conservative, but 17 TF pairs are highly dynamic between GM12878 and K562. Using TF or HM association strength with gene, we investigated the regulatory potency of TF/HM in predicting gene expression level and their dynamics variation between cell lines. Those studies provided a detailed correlation analysis of the 66 regulatory factors, and new insight for the cooperation of TFs and HMs on gene expression. The results are helpful in understanding interaction patterns of TF/HM as well as their cell line specificity in the gene expression and regulation process.

In short, we integrate ChIP-seq and RNA-seq data to explore TF/HM interactions related with gene expression and further their dynamics across cell lines. These researches are helpful for the further study of the interaction for various factors in the gene expression and regulation process. In methodology, we propose a set of novel indexes to study the interaction among TF/HM, and provide new insight for the dynamic regulation of TFs and HMs on gene expression. We constructed a SVM model for the identification of gene expression level using each TF or HM as the single predictor. By listing TFs and HMs based on the predicting power, we can further investigate the regulatory potency of TF and HM.

## Methods

### Matched RNA-seq and ChIP-seq data

The genomic coordinates of the Hg19 human Refseq genes were downloaded from UCSC (http://genome.ucsc.edu/cgi-bin/hgTables). We excluded overlapping gene transcripts in 20 kb region upstream and downstream of TSS and leaved a set of 9555 genes for analysis. In GM12878 and K562, ENCODE Consortium (https://www.encodeproect.org/) provided the comprehensive ChIP-seq for TFs and HMs and matched RNA-seq data. The ChIP-seq data of 55 TFs (narrow peaks format) and 11 HMs (broad peaks format) in common in both cell lines were extracted for the following analysis and calculation. The peak data shows context specific location in whole genome for a specified transcription factor binding or histone modification in a given cell type. This allows us not only to analyze TF/HM co-localization in one cell line but also compare co-localization dynamics across cell lines.

The matched RNA-seq data of GM12878 and K562 were also obtained from ENCODE. Based on the FPKM definition (fragments per kilobase of exon per million fragments mapped), the gene expression levels of 9555 genes were calculated by Cufflinks algorithm [20, 40, 41] according to the RNA-seq expression profiles in two cell lines. Then all genes were divided into 4 clusters by quartile according to the FPKM. The top 25% genes (2389 genes, FPKM≥3.58) and the bottom 25% genes (2389 genes, FPKM≤2.9 × 10^− 5^) were classified as highly and lowly expressed genes, respectively, in GM12878. And the top 25% genes (2389 genes, FPKM≥3.68) and the bottom 25% genes (2389 genes, FPKM≤0.9 × 10^− 5^) were classified as highly and lowly expressed genes, respectively, in K562 (Additional file 1: Figure S2).

### Total difference index

To understand the dynamics of TFs and HMs among cell lines, we focus on their distribution characteristics and differences near TSS. Firstly, a 40 kb DNA region flanking TSS for each transcript was separated into 200 bins. Each bin is 200 bp in size. Then, we obtained 200 bins centered at TSS (20 kb upstream and 20 kb downstream). We assumed that the mid-point of signal peaks is the interaction site between TFs (or HMs) and DNA. For a given TF or HM, we counted the number of peaks in the *j*th bin of the *i*th gene for the *α*th cell line called ${N}_{ij}^{\alpha }$. Then, the signal intensity ${S}_j^{\alpha }$ in each of the 200 bins in the *α*th cell line was calculated with *n* genes by the following formula.

1 $$ {S}_j^{\alpha }=\frac{10^3}{n}\sum \limits_{i=1}^n{N}_{ij}^{\alpha}\left(\alpha =G,K\right) $$

Here *n* equals to 9555. GM12878 is denoted as *G*, and K562 is denoted as *K*.

Next, we defined a total difference index *D*_*signal*_ as follows to investigate the dynamics of TF or HM localization between the two cell lines.

2 $$ {D}_{signal}=\frac{\sum \limits_j{S}_j^G-\sum \limits_j{S}_j^K}{\sum \limits_j{S}_j^G+\sum \limits_j{S}_j^K} $$

And the ratio of the signal intensity between GM12878 and K562 can be denoted by

3 $$ f=\frac{\sum \limits_j{S}_j^G}{\sum \limits_j{S}_j^K} $$

### The overlap ratio and the average overlap ratio

**Fig. 7:**
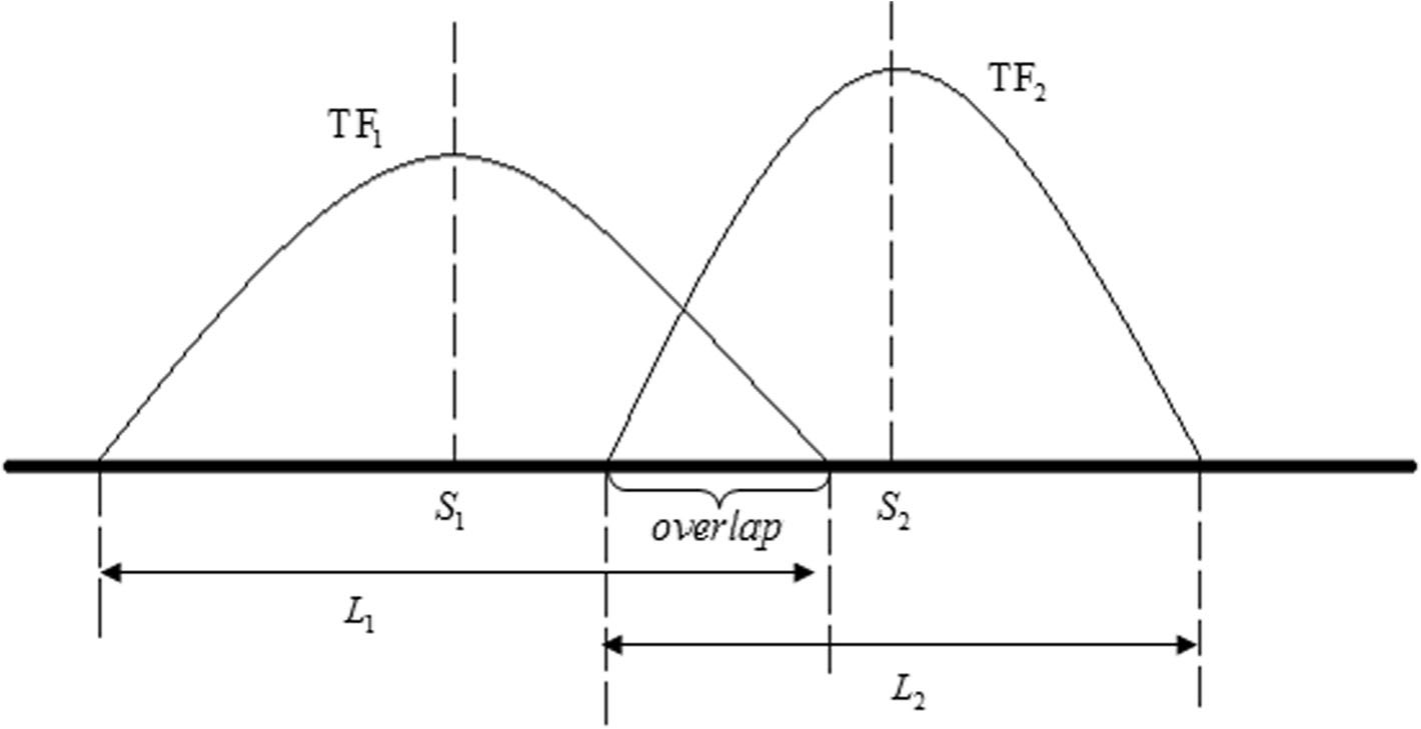
The schematic diagram of the overlap state between TF_1_ and TF_2_. There are two peaks from TF_1_ and TF_2_ respectively. *L*_1_ and *L*_2_ are the peak widths, and *S*_1_ and *S*_2_ are the peak centres of TF_1_ and TF_2_ respectively

For further investigating the potential interaction among TFs, the genome-wide overlap degree of each TF pair was analyzed. As shown in Fig. 7, the overlap state is estimated by the following formula,

4 $$ \left|{S}_1-{S}_2\right|<\frac{L_2+{L}_1}{2}. $$

Here *L*_1_ and *L*_2_ are the peak widths, and *S*_1_ and *S*_2_ are the peak centers of TF_1_ and TF_2_ respectively. Then, the overlap state is encoded into binary states (equal to 1 if formula (4) is holds; otherwise 0). We defined the overlap ratio as follows,

5 $$ {R}_o=\frac{2n}{N_1+{N}_2}. $$

Where *n* is the number of the overlapping peaks between two TFs, and *N*_1_ and *N*_2_ refer to the total peak number of TF_1_ and TF_2_ respectively. The value indicates the genome-wide co-localization degree of two TFs. We assume that the cooperativity and the co-localization degree are closely related.

Given a transcription factor, such as TF_1_, with *m* binding peaks (*P*_1_,*P*_2_,…*P*_*m*_), we investigated the overlap state of each peak with other TFs’ peaks and obtained a vector $\overrightarrow{X}=\left\{{x}_1,{x}_2,\cdots {x}_m\right\}$ for *m* peaks. And *x*_*i*_(*i* = 1, 2, ⋯, *m*) is the number of transcription factors which have at least one peak overlapped with the *i*th peak of TF_1_ (Additional file 1: Table S3). We defined the average overlap ratio *R*_*av*_ as follows,

6 $$ {R}_{av}=\frac{1}{m}\sum \limits_{i=1}^m\frac{x_i}{N} $$

Here, the total number of other TFs is represented by N, and it is 54 in this study. The parameter *R*_*av*_ indicate the extent of potential interaction for this TF with other TFs.

### TF or HM association strength to target gene

Ouyang et al. [21] defined TF association strength (TFAS) which integrated all the peak intensity of a TF by considering their proximity to a gene. Let *g*_*k*_ be the intensity of the *k*th binding peak of TF_*j*_ or HM_*j*_ and *d*_*k*_ be the distance between the TSS of gene *i* and the *k*th binding peak, the TFAS of TF_*j*_ or HM_*j*_ on gene *i* is expressed by

7 $$ {A}_{ij}=\sum \limits_k{g}_k{e}^{-{d}_k/{d}_0} $$

Here we sum all the binding peaks(*k*)of a given TF or HM within a sufficiently large distance (20 kb upstream and 20 kb downstream of TSS) of gene *i*. We set *d*_0_ equal to 2 kb which depends on the distance distribution of TF signal peaks.

### The strength correlation of TF pairs around TSS

TFAS is designed to measure the strength of a TF regulating its target gene. Here, we introduced TFAS to analyze the potential interaction between transcriptional factors in TSS region. For *n* genes, we calculated the TFAS value of 55 TFs based on their signal peaks in 40 kb region centered on TSS. Then, the potential interaction of a pair of TFs was estimated by Pearson correlation coefficient (PCC) of two sets of TFAS values. For example, the PCC between TF_x_ and TF_y_ was calculated as follow

8 $$ {p}_{x,y}=\frac{\sum \limits_{j=1}^n\left({x}_j-\overline{x}\right)\left({y}_j-\overline{y}\right)}{\sqrt{\sum \limits_{j=1}^n{\left(x-\overline{x}\right)}^2\sum \limits_{j=1}^n{\left(y-\overline{y}\right)}^2}}. $$

Where *X* : {*x*_1_, *x*_2_, …, *x*_*n*_} and *Y* : {*y*_1_, *y*_2_, …, *y*_*n*_} are the vectors of the TFAS values for TF_*x*_ and TF_*y*_, $\overline{x}$ and $\overline{y}$ are the means of *X* and *Y*. The PCC values (−1 ≤ *p*_*x*, *y*_ ≤ 1) provided a new criterion to explore TF pair’s potential interaction. The higher PCC, the stronger interaction tendency.

### SVM classifier

We used libSVM to predict the gene expression level [37] using the TFAS value of individual TFs (or HMs) and their combinations as feature. We predict the binary expression level of gene (high/low) and analyze and compare the predictability or contribution of TF and HM on gene expression in GM12878 and K562. A comprehensive list of 66 factors including 55 TFs and 11 HM were used.

### Prediction evaluation

According to 5-fold cross-validation, 9555 genes were randomly partitioned into 5 sets with equal sizes. A single set is retained as the validation data for testing the model, and the remaining 4 sets were used as training data. The process is repeated 5 times, with each of the 5 sets used exactly once as the validation data. The 5 results were averaged to produce a single estimation. Finally, the prediction accuracy are estimated by sensitivity, specificity, and accuracy as follows.

9 $$ {S}_n=\frac{TP}{TP+ FN},{S}_p=\frac{TN}{TN+ FP}, Acc=\frac{S_n+{S}_p}{2} $$

Here, *TP* and *TN* are the number of true positives and true negatives. It means genes with high (low) expression level are predicted correctly. *FN* and *FP* are the number of false negatives and false positives. It means that genes with high (low) expression level are predicted incorrectly.

## Additional file

Additional file 1: **Table S1.** The brief introduction of two cell lines. **Table S2.** Transcription factors associated with cancer in the 55TFs. **Table S3.** The definition of the average overlap ratio for TF1 with m peaks. **Figure S1.** The overlap ratios of 11 HMs with 55 TFs in GM12878. **Figure S2.**The distribution of gene FPKM values in GM12878 and K562 (DOCX 52 kb)

## Abbreviations

FPKM: Fragments per kilobase of exon per million fragments mapped
HM: Histone modification
PCC: Pearson correlation coefficient
TF: Transcription factor
TFAS: TF or HM association strength
TSS: Transcription start site
