# Supplementary material for: Revealing transcription factor and histone modification co-localization and dynamics across cell lines by integrating ChIP-seq and RNA-seq data
Source: BMC Genomics. 2018 Dec 31;19(Suppl 10):914. doi: 10.1186/s12864-018-5278-5 (PMC6311957; doi:10.1186/s12864-018-5278-5)
Supplement: Supplementary file 1 — Table S1. The brief introduction of two cell lines. Table S2. Transcription factors associated with cancer in the 55TFs. Table S3. The definition of the average overlap ratio for TF1 with m peaks. Figure S1. The overlap ratios of 11 HMs with 55 TFs in GM12878. Figure S2.The distribution of gene FPKM values in GM12878 and K562 (DOCX 52 kb) [file 12864_2018_5278_MOESM1_ESM.docx]

**Additional file 1**

Table S1. The brief introduction of two cell lines

| **Cell** | **Description** | Lineage | Tissue | Karyotype | Sex |
| --- | --- | --- | --- | --- | --- |
| GM12878 | B-lymphocyte， lymphoblastoid， International HapMap Project - CEPH/Utah - European Caucasion， Epstein-Barr Virus | mesoderm | blood | normal | F |
| K562 | Lozzio from the pleural effusion of a 53-year-old female with chronic myelogenous leukemia in terminal blast crises." - ATCC | mesoderm | blood | cancer | F |

Table S2. Transcription factors associated with cancer in the 55TFs

| **Family** | **Full Name** | **Members (Official Gene Symbols)** |
| --- | --- | --- |
| AP1 | Activator Protein 1 | FOS，FOSB，JUN，JUNB，JUND |
| ATF | Activating Transcription Factor | ATF1-7 |
| BCL | B-cell CLL/lymphoma | BCL3，BCL6 |
| CEBP | CCAAT/enhancer binding protein | CEBPA，CEBPB，CEBPD，CEBPE，CEBPG |
| CREB | cAMP responsive element binding protein | CREB1-5，CREM |
| E2F | E2F transcription factor | E2F1-7 |
| EGR | early growth response protein | EGR1-4 |
| ELK | member of ETS oncogene family | ELK1，ELK3，ELK4 |
| ETS | ETS-domain transcription factor | ETS1，ETS2，ETV4，SPI1 |
| MYC | myelocytomatosis viral oncogene homolog | MYC |
| SP | sequence-specific transcription factor | SP1-8 |
| STAT | signal transducer and activator of transcription | STAT1-6 |
| USF | upstream stimulatory factor | USF1，USF2 |

Table S3. The definition of the average overlap ratio for TF1 with m peaks

| *TF_1_* | *TF_2_* | *TF_3_* | *…* | *TF_55_* | *X* | The average overlap ratio |
| --- | --- | --- | --- | --- | --- | --- |
| *P_1_* | 1 | 0 | … | 1 | *x_1_* | *$(\frac{x_{1}}{54}+\frac{x_{2}}{54}+\frac{x_{3}}{54}+...+...\frac{x_{m}}{54})/m$* |
| *P _2_* | 0 | 1 | … | 0 | *x_2_* |  |
| *P_3_* | 1 | 1 | … | 1 | *x_3_* |  |
| *…* | … | … | … | … | *…* |  |
| *P_m_* | 0 | 1 |  | 1 | *x_m_* |  |

Figure S1. The overlap ratios of 11 HMs with 55 TFs in GM12878

Figure S2.The distribution of gene FPKM values in GM12878 and K562
